# On the Effects of Alcohol, or Spirit-Drinking, in Diseases of the Liver

**Published:** 1861-08

**Authors:** W. R. Basham

**Affiliations:** Physician to the Hospital


*On the Effects of Alcohol, or Spirit-Drinking, in Diseases of the Liver. Delivered at Westminster Hospital.* By W. R. Basham, M. D., Physician to the Hospital.

Gentlemen:—Of the penalties which attach to the improvident and excessive use of fermented drinks, none are so certain and determinate as disorders of the liver. The effects produced on the nervous system by the habitual and immoderate indulgence in spirituous liquors may vary in different individuals; but the effects on the liver, if not identical in all, differ only in degree and intensity. Of twenty persons addicted to intoxication, two may suffer from delirium tremens—for it by no means follows that all will; but not one will escape the effects of disordered liver function, and ultimate disorganization of this important viscus. The study of these disorders, then, is of vast importance to the student, for they do not always present the same well-marked pathognomonic symptoms which are found in diseases of the lungs or heart, and the inexperienced may not so readily seize hold of the key to the right interpretation of the symptoms. The student will do well, therefore, to watch and note carefully those which are the most characteristic. The following, which have been recently under treatment, have been selected as typical examples of hepatic engorgement, or what may be more expressively designated as chronich hepatitis, from the abuse of fermented drinks. Before detailing these cases, let me remind you that this form of liver disease, if unchecked, invariably leads to cirrhosis, or hobnailed liver.

*Hepatic Dropsy and Death.*

Michael S——, aged forty-six, was admitted Feb. 14th, 1860, having, a day or two previously, vomited up a quantity of blood. He complained of pain and uneasiness in the right hypochondrium, as well as at the pit of the stomach. There were loss of appetite, retching after food of any kind, and for many months past the stomach had been very irritable, and he had vomited on first rising in the morning. There was faint lemon-colored tinge of the skin, and the conjunctivæ were yellow-tinted, scarcely enough to be called jaundiced. The abdomen was hard and tense, particularly in the region of the liver. The extent of this organ, as indicated by percussion, was beyond the natural limits. Some tenderness was experienced at the margin by pressure; the epigastric region was also full and prominent, and sensitive to pressure; the bowels were tympanitic, and he suffered much from flatulence. No fluid could be detected in the abdominal cavity. He has not been able to recline on the left side for many weeks, and sleeps only on the right; if he turns to the opposite side, a dragging, uneasy, and painful sensation is produced, which lasts as long as he remains in that position. The urine has been scanty, with a pinkish deposit, for some time past, and the dejections pale yet oftemtimes black and dark. He has vomited blood more, than once, but never in such quantity as just before admission. He has had jaundice, and symptoms something like the present about twelve months since. His employment has been various, principally on the river, and he appears to have indulged for years in spirits, whenever he could get them. Rum seems to have been his favorite spirit, and he never began work in the morning without a quartern. Without giving credit to him for the quantities he states that he has taken daily, there can be no doubt, that though not a drunkard, he has habitually, on an empty stomach, consumed spirits, to the detriment if not destruction of his liver. For weeks together, the stomach has not been able to retain solid food, and it is not a little singular upon how small a quantity of solid or nutritious food these spirit-drinkers sustain their physical energies, and are able to continue their daily labor. They will often tell you that for weeks they have not eaten a piece of bread the size of their hand, or meat beyond a mouthful or two. The stomach rejects almost everything it receives, except the fiery fluid, which it craves.

The characteristic symptoms, then, were the irritable stomach, frequent retching, and rejection of solid food; heavy dull aching weight in the right hypochondrium, aggravated by lying on the left side; an attack of hæmatemesis, and an icteritic tint of the skin and conjunctivæ. He was ordered blue pill and colocynth for a purge, with an aloetic draught in the morning; and he was to take, three times a day, the decoction of dandelion, with ten grains of the hydrochlorate of ammonia, and ten of the sesquicarbonate of soda, with each dose. He was cupped to four ounces over the region of the liver. The bowels acted freely.

Two days after admission, the irritability of the stomach ceased, and he was able to retain some farinaceous food. The tension, however, and pain in the hypochondriac region were unabated. The dejections were slightly more healthy in appearance; the urine became clear, and the pink lithates disappeared, and its specific gravity was 1017.

On the 20th, six days from admission, the dejections are reported to have a well-marked bile tinge in them, and th patient began to experience a relish for food. There had been no vomiting since admission. The hypochondriac region continuing very tense and hard, and the dragging uneasiness when resting immediately on the left side suffering no diminution, the hepatic region was directed to be painted with the strong compound tincture of iodine.

On the 29th, a fortnight after admission, the report states a manifest improvement. The appetite had become natural, and the patient expressed a relish for food which he had not known for the last two years. As often happens in these cases, the desire for food often exceeds the power of the exhausted stomach to digest easily what is received, and distressing flatulence often marks this period of the case. A cautious selection of food, both as to quality and quantity, and the employment of carminatives, will successfully relieve this symptom. The aromatic spirit of ammonia and ginger were added to the medicine. Several applications of the iodine paint to the right side were followed by marked improvement in the condition of the patient. For several days the lemon-colored tinge of the skin had disappeared.

On examining the abdomen on the 2nd of March, sixteen days from admission, the surface-walls of this cavity no longer presented the hardness or fullness formerly noticed. Pressure was more easily borne, and the area of hepatic dullness was certainly within narrower limits, although still much beyond what would mark a healthy organ. No difficulty was felt on lying over on the left side, and the dragging pain had completely subsided. The bowels required management, for they were apt to get torpid; but small doses of colocynth pill, with one or two grains of blue pill, regulated this function sufficiently well. The last report stated that the dejections were assuming a natural color.

Do not suppose that because all these symptoms have thus far proceeded most satisfactorily, that this patient's liver will return to a sound and healthy state. I fear this is too much to expect. The limits of hepatic dullness are far beyond what they should be, and any rapid shrinking of this extended area would only imply contraction and atrophy of the organ, to be quickly followed by ascites, and other evidence of obstructed portal circulation. The engorged state of the liver—the chronic inflammation as some would term it—has been relieved; but I fear the man's habits have extended over too great a period for us to hope that the liver has only been functionally deranged. By his own statement, as well as by the records of the hospital, he has suffered probably more than one similar attack; we know for certainty of one.

Now these attacks of chronic hepatitis, except in the mildest form, terminate in the formation of organizable matter in the liver, temporary increase of size, and eventually in cirrhosis or gradular liver more or less pronounced. I think my opinion will be borne out by the experience of others, that in a spirit-drinker, who has suffered from hæmatemesis, with symptoms of chronic inflammation of the liver, such as have been detailed, the liver has suffered an amount of damage which may, by altered habits and appropriate treatment, never extend beyond the limits of the first attack, but the effects of which can never be removed; the foundation is thus laid for a process of disorganization, which continues with a tardiness or rapidity directly proportioned to the abstinence or indulgence in spirituous liquors. Let me direct your attention to the nature of this morbid process, which so often speedily leads to fatal disorganization of this organ. To comprehend this process the more perfectly, you should be told that the disease is, for the most part, developed in spirit-drinkers. Persons of vitiated and intoxicated habits, who drink beer or wine, are not so prone to liver disease. In them, it may be said that the nervous system is more prone to suffer. Consider the circumstances under which the alcoholic stimulus is received in either class. The spirit drinker, for the most part, takes the raw undiluted spirit, or dram, on an empty stomach. Fasting in the morning, and at intervals through the day, the dram takes the place of the ordinary meal; for the spirit has essentially deprived the palate of its desire and the stomach of its power to digest solid food. It is well known that spirits completely suspend the secretion of the gastric fluid. The spirit thus received by the empty stomach passes at once, by absorption, to the portal vessels, and in its admixture with the blood, while passing through the liver, it appears slowly and most insiduously—for it is only when the taking of spirits fasting has become grafted into a habit that these morbid effects follow—to set up a sluggish and chronic form of inflammation along the portal canals throughout the liver. At this period the state of the liver and portal system of vessels is one of engorgement or blood stasis. This continuing, the usual effect follows—the formation and deposit of lymph in the areolar structures surrounding the portal vessels, or, in other words, in the portal canals. It is this engorgement and subsequent effusion of lymph which constitutes the state of chronic inflammation, and gives rise to the fullness and tension, and sense of weight and dragging in the right hypochondriac region.

Now this is the state in which we found the liver of this patient on his admission. In his case it was not the first attack of engorgement he had suffered, and the symptoms were, therefore, the more strongly pronounced. And that something more than congestion or hyperæmia of the liver existed, is inferred from the hæmatemesis, which never occurs till after considerable obstruction and obliteration of the portal vessels have been accomplished. Now, you will probably ask, why is it that similar effects do not follow the drunkard from other forms of alcoholic stimuli? First, in beer the spirit is largely diluted, and is, moreover, combined with glucose, and oftentimes with small quantities of unchanged dextrine and other organic matters; on all these the stomach exerts its solvent and digestive action. The alcohol thus is not so immediately carried into the circulation, nor does it exercise so poisonous an influence on the blood. Though wine contains far more alcohol than beer, yet those who drink wine for the most part consume it after meals; it is thus received during the primary stages of digestion, and its chief injury consists in retarding or weakening the digestive process, and its alcohol does not reach the liver under the same deliterious conditions as crude undiluted spirit. Here, then, is the explanation why raw spirits so frequently lead to atrophy and shrinking of the liver, and why beer or wine drinkers are comparatively more exempt. The state of the liver of this patient, then, at the present period, is that of augmented bulk from the deposit of adventitious tissue in the portal canals. The first stage of scirrhosis is, therefore, one of enlargement, from the effects of a chronic or subacute inflammatory process, which is succeeded by a deposit in the channel of the portal vessels. The subsequent shrinking or atrophy of the organ is explained by the contraction of the fibrous tissue into which the lymph is converted that was formed during the inflammatory stage. Nor is it alone by the shrinking of the adventitious tissue that the atrophy and nodular or hobnail surface is produced. Dr. Budd, in his excellent and valuable work "On Diseases of the Liver," shows that this contraction of the tissue compresses, and even obliterates, the smaller portal veins; and the parts which they supplied become atrophied or destroyed, as any organ would be the vascularity of which was diminished, and the secreting power of which was also proportionately decreased. But, in the case before us, this stage has not yet arrived; for on examining to-day (March 15th,) the liver, when mapped out by percussion, occupies an area greater than natural; so that the organ at present represents the stage of recent deposit of lymph before its more fluid parts have been removed, or it has become converted into fibrous areolar tissue.

The chief symptom under which the patient at present labors is imperfect digestion, expressed by flatulence immediately after taking food, and a sense of uneasiness and oppression during the primary stage of digestion. This arises from the imperfect formation of the gastric juice, originating doubtless in the impeded circulation through the liver. Whatever hampers the return of blood from the stomach through the portal system causes a stagnation or remora of blood in the gastric mucous membrane and follicles, and such a retardation impedes the secretion or formation of a perfect gastric juice. If the food, on being received by the stomach, is not immediately acted on by a well-elaborated gastric solvent, it quickly undergoes molecular changes, which are more akin to chemical than to vital processes. Various gaseous products are formed, which distend the walls of the stomach, and still further impede the formation of the true gastric acid. The patient feels oppressed and heavy, and full and distended. The direction from which relief of these symptoms is to be expected is from an increased activity of the hepatic function, and this is often readily obtained at this stage of the disease by small doses of blue pill and ipecacuanha. Two grains of blue pill and one of ipecacuanha should be given every night, or alternate nights, for several doses. The object is not to salivate· but to increase the activity, if possible, of the portal circu, lation through an increase in the secretion of bile. The patient has always improved after a few such doses. He has throughout the whole course of treatment been taking the liquor taraxaci and the hydrochlorate of ammonia. The former is a popular remedy in all cases of hepatic derangement; but of the latter remedy I am inclined to think that its efficacy in visceral disorders is not so generally known. It does not appear to possess very strong cholagogue properties; but its action seems to promote the removal, or at any rate to diminish the condensation, of adventitious tissue, formed by acute or subacute inflammation.

As this case presents only the symptoms of the early stage, the period of engorgement and of adventitious deposit, antecedent to a further disorganization of structure, leading to cirrhosis or hobnail, liver and as the conditions —such as ascites—which characterize that advanced stage are not before us, I shall postpone to a future occasion some practical remarks as to the nature of those pathological changes, as well as to the origin and progress of the ascites which constitutes the last stage in the progress of the dram-drinker's liver. Your attention must be called to the advantage which follows local depletion in these cases. A few ounces of blood taken by the cupping glass over the hepatic region, and followed by brisk mercurial and colocynth purgatives, is a course of treatment which experience justifies. You will find, however, that the advantages of cupping are proportioned to the period of the disorder in which it is performed. It is only when you have signs of active hyperæmia that benefit can be expected. After that period has passed, and when the portal canals may be the seat of recent deposit, counter-irritation affords the best prospect of a remedial effect. The application of the iodine paint, as it is sometimes called (the compound tincture of iodine)—covering the entire hepatic region with this solution, is a judicious plan of treatment, and one the effects of which are illustrated in the case before us.

I must now call your attention to another case of hæmatamesis occuring in the patient in the next bed, which will still further exemplify the progress of these hepatic disorders generated by the vicious habit of dram-drinking. The hepatic disease is not so advanced as in the last case, nor is it, I hope, of so serious a character.

Samuel M——, fifty-six years of age, of a stout, somewhat obese, habit of body, was admitted Feb. 24th, having for several days before admission vomited a considerable quantity of dark-colored clotted blood. He thinks that upon one occasion as much as three pints were vomited. He appears like one who had suffered from severe hæmorrhage; his cheeks are blanched, lips white, inner edge of eye-lids exsanguineous, and tongue pale and flabby. The epigastric region was full, and there was some tenderness on pressure. He felt a dull, heavy, dragging pain in the right hypochondrium. For many days the dejections have been very dark and discolored. The urine was very high-colored, specific gravity 1018, and on cooling there was a plentiful deposit of pink urates. These pink urates are very common in all these hepatic disorders. On examining the abdominal region, some degree of fullness was observed in the right hypochondrium; the margin of the liver extended two fingers' breadth lower down than was natural and dullness extended upwards beyond the usual limits. He stated that he suffered a similar attack of blood-vomiting last summer. These attacks were always preceded by loss of appetite, and nausea and retching on first arising in the morning. He has had little or no relish for solid food for a year or two. His occupation in life has been that of a gentleman's servant, principally in the capacity of butler, but, having left service, he enjoyed remarkably good health till he was tempted to take a public house in a village on the banks of the Thames. Here either the temptations were too great, or the customs of the trade, as some will insist, led him to the pernicious habit of dram-drinking; about three half-quarterns he confessed to have been his daily allowance. Now, it is perfectly immaterial whether this was the maximum or the minimum of his daily potations. It suffices that he fell into the habit of taking spirits on an empty stomach—sometimes undiluted, sometimes otherwise,—and that this habit was followed by defective digestion, loss of appetite, nausea, vomiting, and subsequently violent attacks of hæmatemesis. In this case there was no jaundice, no tinge of yellow—not the faintest—of the conjunctivæ, and there was no uneasiness experienced on lying on the left side. The symptoms were altogether milder than in the last case; but then the habits of this patient had not existed for more than a year or two, and, fortunately for his future health, had been effectually checked, and his exposure to temptation suddenly withdrawn, by the abrupt termination of his occupation, his relentless landlord thinking that another tenant might sell more and pay better. A brisk purgative of calomel and colocynth, followed by a warm aloetic draught in the morning, operated favorably. He was ordered the liquor taraxaci, sesquicarbonate of soda, and ammonia.

Four days after admission, he is reported as much better; no vomiting, or even nausea; the stomach retains simple articles of nutrition—beef-tea and arrow-root. On the sixth day, as some fullness and weight was still experienced in the right side, he was ordered two grains of blue pill and one of ipecacuanha at bed time for four nights. The alvine dejections assumed gradually an improved appearance, and the urine became quite clear, natural, and pretty abundant; the tongue was clean, although pale; the epigastric tenderness had disappeared, and the patient began to crave for food—to feel hungry—a most important and a most encouraging symptom in these cases. After he had continued a fortnight under treatment, the chief indication became to supply, if possible, the great blood loss he had sustained, at the same time taking care that the weak digestive powers and the impeded hepatic function were not too much overtaxed. Gradually he was able to take and relish the full-diet allowance of the hospital. To regulate the bowels, gently stimulate the flow of bile, and help to promote the blood-forming powers, he was ordered each night eight grains of aloes and myrrh pill, with half a grain of the sulphate of iron. Under this treatment, he progressed so favorably, that on March 10th he left the hospital in a condition very favorable to the arrest of any further disorganization of the liver, provided he avoided the injurious habit which had brought on this serious attack. A complete recovery may be expected in this and similar cases in the *earliest* stage, where the habits of the patient, either by the force of resolution or of necessity, are ultimately regulated by the dictates of sobriety and self-denial.

I will relate a case which occurred in private practice, to which I was called by my friend, Mr. Geo. Pearse, and which will teach you that something, and indeed a great deal, may be done even in cases where the disorganization has reached a point in which obstruction to the portal circulation was sufficient to produce extensive ascites.

A man about fifty years of age, whose drink for fourteen or fifteen years had been gin, spending, according to his own account, fourteen shillings weekly in drams, suffered, when he first came under Mr. Pearse's care, from ascites and some anasarca of the lower extremities, gradually and almost imperceptibly increasing. The propriety of tapping became the question to be solved. I found him propped up in bed, the horizontal position being impossible. The abdominal walls were distended till the surface of the skin glistened, it was so smooth. The evidence of fluid by succussion was undoubted; the superficial abdominal veins were much enlarged, and anastomosed upwards freely into the branches of the external mammary and intercostal vessels. There was considerable emaciation of the trunk and upper extremities—always the more striking in these cases from the distended abdominal walls and roundness of texture of the lower extremities from the existence of anasarca. The breathing had become difficult at times, but there was not urgent dyspnœa. The hearts sounds were quite natural. The urine was very scanty, high-colored, and presented a copious deposit of pink urates. His history was that of a dram-drinker. Morning, noon, and night, the "half-quartern" worked its insidious and baneful affects: loss of appetite, nausea, morning sickness, inability and absence of all desire for solid food, and at length one or two attacks of hæmatemesis, plainly told the progress the disease was making. Yet, strange to say, these signs carried no warning with them. And thus it ever is: the bane is mistaken for the antidote; the poison is accepted as the remedy; and not, perhaps, till unmistakable signs of dropsy, sufficient to interrupt the patient's employment, have shown themselves, does he seek medical advice, and learn, when it is too late, that his disease is of his own creating, and that the habits of years have been the fatal source of his premature decay. This patient had been very judiciously treated with diuretics and elaterium, but without producing any effect on the dropsy, which seemed so much on the increase that the only relief expected was from tapping. But as this operation should never be resorted to till all other remedies have failed, and there exists such impediment to the respiration by the upward pressure of the fluid on the diaphragm that life is jeopardized, I advised that trial should be made of other diuretics and other purgatives. An examination of the urine having satisfied me that no renal complication would interfere or prohibit the use of these remedies, he was ordered the tincture of digitalis, with half-drachm doses of the sweet spirits of nitre, and ten grains of the ascetate of potass, every four hours; and the bowels were to be kept active by the compound jalap powder, in drachm doses every morning. The question of tapping was postponed for several days, that it might be seen what relief was to be obtained from this treatment. In two days the breathing was found to be materially relieved. The powders had acted briskly, and already the kidneys had begun to excrete more freely, for the urine had become abundant and clear. He had not slept for many nights before this, from the distress to his breath, but he now had several hours quiet refreshing sleep. In eight days a great improvement became apparent. He was able to take solid food; all anasarca from the lower limbs had disappeared; the abdominal walls became much softer; he could breathe freely, and had natural and refreshing sleep. The last I heard of him was, that the abdominal parietes had decreased to their natural limits, with obscure sense of fluid in small quantity.

I have had no opportunity of examining this patient since my first visit to him. I am indebted for these particulars to Mr. Whitmarsh, who tells me that the patient is able to employ himself in some light occupation with advantage, and that he feels better than he has done for years.

I have mentioned this case to you, less for the purpose of explaining the pathology or symptoms of scirrhosis with dropsy, than for illustrating what may be done, even in unpromising cases, by a well-directed and persevering plan of treatment, Had this man been tapped, he could never have reached his present state of improvement.—*London Lancet.*

